# Supplementary material for: The genome sequence of the July Highflyer, Hydriomena furcata (Thunberg, 1784)
Source: Wellcome Open Res. 2023 Oct 26;8:496. [Version 1] doi: 10.12688/wellcomeopenres.20182.1 (PMC11134148; doi:10.12688/wellcomeopenres.20182.1)
Supplement: Peer review history

# Reviewer report: Reviewer response for version 1

**DOI:** 10.21956/wellcomeopenres.22345.r73853

**Published:** 2024-07-15

**Authors:** Jin-Wu Nam

**Affiliations:** 1 Hanyang University, Seongdong-gu, Seoul, South Korea

1. They presented a genome assembly of  July Highflyer using Pacbio long-read HIFI sequencing data and Hi-C data, acquiring a pretty high-quality of genomes as well as the mitochondrial genome. In addition, they annotated protein-coding genes using short and long-read RNA-seq data , identifying 17,324 protein-coding genes in a draft genome.  Overall, they presented a high-quality result and well-described methods. 
2. It would be great to have a comparative analysis of assembled genomes of host and mitochondria with closed-related specie's genomes as well as protein-coding genes, which will provide interesting insights.  In addition, I suggest that authors can examine repeat elements in genomes and how much and what kinds of repeats are prevalent.

Are sufficient details of methods and materials provided to allow replication by others?

Yes

Is the rationale for creating the dataset(s) clearly described?

Partly

Are the datasets clearly presented in a useable and accessible format?

Yes

Are the protocols appropriate and is the work technically sound?

Yes

Reviewer Expertise:

Genome assembly, Structural variations

I confirm that I have read this submission and believe that I have an appropriate level of expertise to confirm that it is of an acceptable scientific standard.

# Reviewer report: Reviewer response for version 1

**DOI:** 10.21956/wellcomeopenres.22345.r84783

**Published:** 2024-07-15

**Authors:** Michael Hiller

**Affiliations:** 1 Senckenberg Nature Research Society, Frankfurt Am Main, Germany

- This data note reports a high-quality primary assembly of the July Highflyer.
- The genome was generated from PacBio HiFi data, polished with 10X reads and scaffolded with HiC data.
- The assembly has a very high base accuracy and BUSCO completeness, and almost all of the contigs are assigned to chromosome-level scaffolds. The mito genome was also assembled and genes were annotated in both the mito and nuclear genome.
- All methods and procedures are clearly described.
- The assembly contributes to the genomic resources available for lepidoptera.

I have two minor comments:

- I wonder why polishing with the 10X data was necessary, as one often / mostly achieves a QV > 60 with HiFi reads alone and this study generated sufficient (42X) HiFi coverage.
- In Figure 1, it would be helpful to note whether the missing piece of the left wing is a natural feature or damage caused by sampling the individual.

I am also wondering if the wing color variation can be illustrated in case images are available.

Are sufficient details of methods and materials provided to allow replication by others?

Yes

Is the rationale for creating the dataset(s) clearly described?

Yes

Are the datasets clearly presented in a useable and accessible format?

Yes

Are the protocols appropriate and is the work technically sound?

Yes

Reviewer Expertise:

comparative genomics, genome assembly, methods

I confirm that I have read this submission and believe that I have an appropriate level of expertise to confirm that it is of an acceptable scientific standard.

# Reviewer report: Reviewer response for version 1

**DOI:** 10.21956/wellcomeopenres.22345.r84779

**Published:** 2024-05-28

**Authors:** Kay Lucek

**Affiliations:** 1 University of Neuchâtel, Neuchâtel, Switzerland

The authors present the chromosome level genome assembly of a male specimen of the July highflyer *Hydriomena furcata*. The assembly consists of 28 chromosomes including the Z chromosome. The assembly is highly complete as revealed by the high BUSCO score but not fully phased. Sequencing and genome assembly follow the current state of the art and use established methods.

 Overall, the presented assembly will be of great value to study genome architecture as well as genome size evolution in Lepidoptera. I particularly appreciate the annotation using RNAseq data, although the specific dataset is not indicated but only indirectly in the referenced BioProject.

Are sufficient details of methods and materials provided to allow replication by others?

Yes

Is the rationale for creating the dataset(s) clearly described?

Yes

Are the datasets clearly presented in a useable and accessible format?

Yes

Are the protocols appropriate and is the work technically sound?

Yes

Reviewer Expertise:

Speciation genomics

I confirm that I have read this submission and believe that I have an appropriate level of expertise to confirm that it is of an acceptable scientific standard.

# Reviewer report: Reviewer response for version 1

**DOI:** 10.21956/wellcomeopenres.22345.r73855

**Published:** 2024-05-16

**Authors:** Rodolpho S.T Menezes

**Affiliations:** 1 State University of Santa Cruz, Ilhéus, Brazil

The manuscript by Boyes and Holland presents a genome assembly from a male of the July highflyer, *Hydriomena furcata *(Thunberg, 1784). The final assembly is 423.3 megabases and most of the assembly is scaffolded into 28 chromosomal pseudomolecules, including the sex chromosome. Overall the manuscript presents an advance in the genomic field of Lepidoptera. This genome information will be interesting for comparative genomics investigation and also for evolution and conservation studies. The methodology is thorough and explained and the figures are well constructed and the manuscript is well written. However, I think the author should compare its results with the assembled genomes from other Lepidoptera species.

 Minor point: Since the author assembled the mitochondrial genome, I think it is interesting to show general (GC/AT content…) mitochondrial information about this species.

Are sufficient details of methods and materials provided to allow replication by others?

Yes

Is the rationale for creating the dataset(s) clearly described?

Yes

Are the datasets clearly presented in a useable and accessible format?

Yes

Are the protocols appropriate and is the work technically sound?

Yes

Reviewer Expertise:

Phylogenomics, Cytogenetics, and Phylogeography.

I confirm that I have read this submission and believe that I have an appropriate level of expertise to confirm that it is of an acceptable scientific standard.
